# The Entire Uterus Torn from a Puerperal Woman without Fatal Consequences

**Published:** 1880-06

**Authors:** 


### THE ENTIRE UTERUS TORN FROM A PUERPERAL WOMAN WITHOUT FATAL CONSEQUENCES.

Dr. Schwartz reports in Speigelberg's *Archiv of Gyneckologie* a case in which the midwife in attempting the removal of a retained placenta, had grasped the inverted fundus, dragged it down, and tore the whole organ from the vagina. Escape of the intestines was prevented by a tampon saturated with salicylic acid. On the fourth day the woman was free from fever, and by the twenty-first had fully recovered. At the latter date exploration of the vagina showed its walls to be perfectly united at its upper extremity. The woman has since then continued in excellent health.—*Berliner Klinische Wochenshrift, No. 3, 1880. Cin. Lancet and Clinic.*

